# Latent Classes of Substance Use in Adolescent Cannabis Users: Predictors and Subsequent Substance-Related Harm

**DOI:** 10.3389/fpsyt.2014.00009

**Published:** 2014-02-07

**Authors:** Jean-Sébastien Fallu, Frédéric N. Brière, Michel Janosz

**Affiliations:** ^1^École de Psychoéducation, Université de Montréal, Montréal, QC, Canada; ^2^Institut de Recherche en Santé Publique de l’Université de Montréal, Montréal, QC, Canada; ^3^School Environment Research Group, Université de Montréal, Montréal, QC, Canada

**Keywords:** cannabis use, substance use, classes, adolescents, substance-related problems

## Abstract

Cannabis use is highly prevalent in late adolescence, but not all users experience significant negative consequences. Little information is available to identify the substance use patterns and risk factors of users who are at greater risk of experiencing negative consequences. In this prospective study, we aimed to empirically identify latent classes of substance use in adolescent cannabis users and to examine how these classes relate to antecedent psychosocial predictors and subsequent substance-related outcomes. The sample was recruited from 68 high schools in Quebec and consisted of 1618 participants who reported using cannabis in grade 10. We used latent class analysis to empirically identify classes of users based on the age of onset, frequency, and typical quantity of cannabis and other substance use, as well as substance mixing behaviors. We then compared classes in terms of (a) sociodemographic and psychosocial predictors in grades 7–8 and (b) substance-related consequences in grade 11. Four distinct classes were identified: Late-Light Users (28%); Late-Heavy + Polydrug Users (14%); Early-Moderate Users (33%); Early-Heavy + Polydrug Users (26%). Late-Light Users reported the lowest levels of substance use, while Early-Heavy + Polydrug Users reported the highest levels. Intermediate levels of substance use were found in the other two classes. Sex, age, delinquency, peer delinquency, school bonding, parental monitoring, and parental conflict all helped to differentiate classes. Class membership predicted substance-related harm, with greater consequences in early- and late-onset heavy using classes. In light of results, in addition to age and sex, screening and intervention for risky cannabis use among adolescents should focus on school bonding in order to target the most risky late-onset adolescents and on peer delinquency in order to target the most risky early-onset ones.

## Introduction

Cannabis is the illicit drug most widely used in adolescence. By late adolescence, cannabis use is a relatively normative behavior. The latest available figures of annual cannabis use by late adolescents in North-America vary from close to 40% in the USA (1) to close to 50% in Quebec, Canada (2). More than 80% of 12 graders find it easy or very easy to have access to cannabis (1). Fortunately, not all cannabis users experience significant negative consequences (3, 4), but some do. A key task of prevention science is to better understand the classes of use, the characteristics of users, and other factors that are related to problematic use. The idea that some use classes are more at risk than others is often put forward (5, 6), but rarely put to the test. A few studies have described typologies of cannabis users, but most have studied clinical samples or have focused mostly on specific problems or solely on cannabis use indicators (6–8). Little information is currently available in the literature to allow identifying and distinguishing between subgroups of cannabis users at higher and lower risk of impairments, which would be critical to improving screening and prevention.

Examining natural heterogeneity in classes of cannabis use may be one helpful strategy to understand why some users experience more problematic consequences than others. Many studies have documented the acute and/or chronic health risks or harms associated with cannabis use. These include cannabis dependence, fatal and non-fatal motor-vehicle accidents under the influence of cannabis, cognitive impairments, respiratory impairments, and the amplification or onset of psychosis, especially in predisposed individuals (9–16). Specifically, studies suggest that several key cannabis use characteristics are most predictive of such harm outcomes. These include frequent (e.g., weekly or more often) or chronic cannabis use, and early-onset of cannabis use (11, 17–20). Because many adolescent cannabis users are polydrug users (2, 21), classes of use also have to take multiple substances into account, including alcohol. Indeed, most cannabis users take it simultaneously with alcohol (22–24), which could be a particular risk for youngsters (25). To our knowledge, no study compared the consequences of empirically derived cannabis use classes in a normative population of adolescents.

Studying psychosocial predictors of heterogeneity in cannabis use classes is also important. This allows identifying factors, which anticipate high-risk substance use patterns vs. low-risk substance use patterns. Many categories of predictors can be useful to predict heterogeneity in patterns. For instance, a recent study by Chabrol et al. (7) applied a cluster analysis to a sample of adolescents cannabis users on the basis of personality traits and found three groups: “ordinary,” below the mean on several measures of personality, “borderline,” with high levels of borderline traits, depressed moods, and social anxiety, and a least prevalent cluster called “impulsive,” which was well above the mean on impulsivity and callous traits but low on other measures. As expected, the frequency of use was higher in the latter two clusters.

In addition to those considered by Chabrol et al. (7), other factors from other domains of influence might be useful and important in predicting heterogeneity. Babor et al. (26) suggested that classification schemes must be multidimensional in order to be useful in predicting outcomes. Accordingly, in order to achieve such classification, one must rely on diverse individual and relational risk factors for substance abuse, use-related problems, as well as substance use patterns. Severity of substance use (27), the level of comorbid psychopathology (28, 29), or delinquency (30) have been common dimensions of classification for adolescent substance abusers, but very few studies have relied on multiple dimensions of risk, use, and related problems. A strong predictor of adolescent substance abuse, family conflict (31), that has been useful in distinguishing “Aggressive/Versatile” delinquents, the most severe and chronic subtype (32), and deviant peer affiliation, which is also a robust predictor of adolescent substance abuse (33) was rarely considered in classification efforts. In sum, relevant factors may include familial conflict and monitoring, peer substance use, school bonding and achievement as well as sex, in addition to the one considered by Chabrol et al. (7), which all proved to be useful in predicting use indicators (31, 34, 35).

Until now, past studies have proposed several typologies based on theoretical grounds and those who used an empirical approach have only shed light on some aspects of reality. Many of these studies focused on alcohol use only (36–38), but some have proposed specific typologies of cannabis users. For instance, among adults, Thomas et al. (39) used epidemiological data to derive a cannabis use typology based on use frequency as well as related harm. Their typology included abstinent and past users. Among users, they proposed three groups: low-risk (26%), moderate-risk (72%), and high-risk/dependent (2%). In terms of empirical studies, Fischer et al. (5) derived a typology of cannabis users, but this study was realized among adults, with a cross-sectional design and focused only on cannabis use indicators (e.g., onset, actual use, daily use, quantity, with whom, medical reasons) to derive their four-group typology: occasional/light use (31.8%), moderate-monthly use (20.2%), moderate-weekly use (25.2%), and near-daily or daily use (22.9%). Reboussin et al. (8) aimed to describe patterns of marijuana involvement during the middle-school years in a sample of African-American adolescents. They also included non-users and used latent class analysis (LCA) on the same cannabis use indicators measured over 3 years. Three classes were identified: little or no involvement (85, 71, 55% in sixth, seventh, and eighth grade, respectively), marijuana exposure opportunity (12, 19, and 26%), and marijuana use and problems (2, 9, and 19%). Another study looked at the typology of cannabis-related harm instead of use indicators in a community sample of 14–24 years old throughout 10 years (6). Four substance categories were considered: alcohol, nicotine, cannabis, and illegal drugs other than cannabis. Four groups were identified: Non-problematic (59.2%); primary alcohol use disorders (14.4%); delinquent cannabis/alcohol DSM-IV-abuse (17.9%); and CUD with multiple problems (8.5%). Another cross-sectional study used cluster analysis on a clinical sample of mostly juvenile justice involved adolescents who sought drug abuse treatment (40). They identified three groups based on individual and family risk factors, associated problems, and severity of substance use: Juvenile Justice Involved Substance Abusers (41%, lowest level of risk but highest juvenile justice involvement); Comorbid Substance Abusers (33%, greatest family risk and individual psychopathology); and Heavy Substance Abusers (26%, serious substance abuse and peer substance use). Variables included were substance use, psychiatric disorders, and legal involvement; peer substance use; family substance abuse; parental psychopathology; and family conflict. This multidimensional typology support the idea that risk factors, associated problems, and substance use severity are all critical in explaining heterogeneity.

Several limitations characterize previous studies. First, the variety of designs used in these studies complicates comparisons between them. Second, few studies have examined subgroups of cannabis users (or heterogeneity in cannabis use) and many of the classification efforts were limited to clinical samples (40). Finally, cannabis use severity and important risk factors, such as peer deviancy, parental monitoring, and school bonding, have typically been omitted in previous typologies.

In this study, we aim to empirically identify subgroups of adolescent cannabis users and examine how these subgroups differ in terms of early risk factors and subsequent consequences. We extend prior work by focusing on a general population of adolescent and by using a comprehensive prospective design. We use latent class analysis (41), which allows assigning individuals to relatively homogeneous classes on a probabilistic basis. An increasing number of recent studies have applied LCA to identify subgroups of substance users (5, 6, 42–46). A main methodological benefit of the LCA approach is that it groups users according to a multiplicity of observed characteristics (e.g., substance use behaviors), as opposed to examining such characteristics separately. This approach is thus a powerful tool to identify and compare multidimensional classes of cannabis users and their associated characteristics.

## Materials and Methods

### Participants

The sample was recruited from 68 high schools in Quebec within the context of the evaluation of the new approaches new solutions (NANS) dropout prevention program (2002–2008) (47). Participants attended secondary schools in disadvantaged communities of the province of Quebec (Canada). NANS schools were selected using stratified random sampling to be representative of all schools in disadvantaged areas of Quebec in terms of geographical location, size, and language (47). Data were obtained via self-reported questionnaires administered in class by teachers supervised by trained and supervised experimenters. Seventy-seven percent of eligible participants provided free and informed consent to participate in the study. All procedures were approved from the Arts and Science Faculty Ethical Review Board at University of Montreal. Participants for this study were a cohort assessed annually from grade 7 to grade 11 (2003–2008). The sample for the present study included all participants who provided information on cannabis use in grade 11 (*N* = 1618). Participants were mostly Quebec-born Caucasians (93%). Other participants were from a diversity of ethnicities. The sample included slightly more females (53%) than males (47%).

Self-reported substance-use behaviors were collected in grade 10. Predictors were considered in grade 7 and 8 and outcomes in grade 11. Available data for outcomes in grade 11 were 61%). Rates of available data for predictors in grades 7–8 ranged from 80 to 99%.

### Measures

#### Substance use behaviors (grade 10)

Substance use measures were mostly taken from the ESPAD questionnaire (48, 49), a European national substance-use survey of a representative sample of high school students. Its reliability and validity have been verified in the content of many methodological studies [see Ref. (49)]. These measures included past-year alcohol and cannabis use frequency. Original items had seven categories: 1: “0”; 2: “1–2”; 3: “3–5”; 4: “6–9”; 5: “10–19”; 6: “20–39”; and 7: “40 or more.” Some categories of the original items were collapsed together, based on their distributions and on the literature (1, 2), in order to limit the number of categories and get clinically significant grouping while avoiding the estimation of a large amount of parameters in the analyses. This resulted in three categories of alcohol and cannabis use frequency: 0: “0–5”; 1: “6–30”; and 2: “31 or more.” We also used the quantity of alcohol consumed in a typical occasion. Again, some categories were collapsed together for the same reasons. This led to a variable in three categories: 0: “0–3”; 1: “4–6”; and 2: “6 or more.” We also added a home measure of the quantity of cannabis taken in a typical occasion and collapsed the categories in two: 0: “1 joint or less”; and 1: “more than a joint.” Binge drinking was also measured using the ESPAD item with collapsed categories: 0: “never”; 1: “1 or 2 times”; and 2: “3 or more times.” Other items were taken from a validated measure of adolescent social and personal adjustment (50): stimulant-hallucinogens use (“never”; “1 or 2 times”; “3 or more times”) as well as two items of alcohol and cannabis use in order to derive alcohol and cannabis use early-onset (grade 8 or earlier). The cannabis and alcohol onset measures are exceptions in the sense that contrarily to other substance use measures, they were derived from grade 7 and 8 items of alcohol and cannabis use frequency. Frequency of tobacco use was measured with a home measure in which categories have been collapsed in the following groups: 0: “never”; 1: less than one per day or “occasional”; and 2: one per day or more or “regular.” Finally, the simultaneous use of alcohol and cannabis was also assessed by a house measure and was coded 0: “never”; 1: “1 or 2 times”; and 2: “3 or more times.” We included this measure as this particular behavior has been associated with negative consequences in previous work (22). All items were referring to the past 12 months except for binge drinking and tobacco use (past 30 days).

#### Substance-related problems (grade 11)

The outcome measure is largely based on the DEP-ADO scale, widely used to screen substance related problems in Quebec (51, 52). This instrument includes 11 items to which we added 3 to include other important substance-related consequences (fights, unprotected or unwanted sex, intoxication in school) for a total of 14 items (α = 0.88). Each item measures the occurrence of different attributed substance-related consequences covering various types of negative consequences, such as legal, school, relational, health, and dependence consequences. Items have been coded 0 (never) and 1 (yes) in accordance with participants’ attributions. A confirmatory factorial analysis (53) indicated that all items could be grouped in a single scale (not shown; results can be obtained upon request).

#### Sociodemographic and psychosocial predictors (grade 7–8)

Sociodemographic and psychosocial factors used to predict latent classes were selected on the basis of existing theoretical and empirical literature (31, 34, 35, 54, 55). Parental monitoring and conflict with parents, delinquent behaviors, peer deviancy, and school bonding and achievement were measured with scales taken from the same questionnaire used for substance use measures, the MASPAQ (50). Parental monitoring was measured with two items asking about parental knowledge of whom their adolescent is with when not at home and where he or she is (“never,” “occasionally,” “often,” “all the time”). Conflict with parents is measured with three items asking about disputes and disagreements with parents with the same item scale. Delinquent behaviors are measured from the presence or absence of a variety of delinquent behaviors (e.g., property crime, fights). Peer delinquency is measured from three items asking about friends’ drug use, and if friends had or could have had trouble with the police. These items respectively have the following scales: “never,” “now and then,” “sometimes,” “often,” “always”; “none,” “one or two,” “several,” “many”; “strongly disagree,” “disagree,” “don’t know,” “agree,” “strongly agree.” School bonding was measured with four items (e.g., I like school; I like what we do in school). The scale is a valence scale with seven categories. Finally, school achievement was measured with two items asking for grades in maths and in French. Depressive symptoms were assessed using the Center for Epidemiologic Studies-Depression (CES-D) scale (56). The CES-D includes 20 items that explore how participants felt or behaved in the past week. The CES-D has been validated for use in French and adolescents (57, 58). Internal consistency was adequate with Cronbach’s alpha ranging from 0.87 to 0.91 across time points. Sociodemographic factors included sex, age, and family adversity as measured by a cumulative index of nine family risk factors (e.g., low parental occupational prestige, low family wealth, parental separation). All previous factors except age and sex were derived from a mean of scores measured in grade 7 and 8.

### Statistical analyses

Latent class analysis was used to identify subgroups of cannabis users. This statistical method aims to identifying the most parsimonious classification of individuals into latent classes by maximizing homogeneity within, and heterogeneity between classes. In order to determine the optimal number of classes, different number of latent classes was modeled starting from 1 (e.g., only one class of cannabis users), then 2, and so on until we reach an optimal solution. Different criteria were used to select the most appropriate model (59). These criteria included the following information criteria: deviance, the Akaike information criterion (AIC) (60), the Bayesian information criterion (BIC), and the sample-size adjusted Bayesian information criterion (SSBIC) (61), to compare the relative fit of solutions. Better fitting solutions are reflected in lower values on the indices. We also considered likelihood ratio tests, including the Vuong–Lo–Mendell–Rubin and Lo–Mendell–Rubin adjusted likelihood ratio tests – ALRTs (62). ALRT tests are adequate for non-nested mixture models and test the significance of the difference in fit between two models with a one class difference. We also considered the recommended Bootstrapped Likelihood Ratio Test [BLRT; (63)]. The criterion for significance was α < 0.05. We also relied on entropy, which is indicative of the degree of homogeneity within and independence between classes (60). Elevated scores of entropy indicate high independence and little spillover between classes. Furthermore, we examined the substantive interest of each model by evaluating how solutions compare with theoretical and empirical knowledge. Finally, although we selected a solution based primarily on unconditional models, we also investigated all solutions with predictors to determine whether all classes could be meaningfully differentiated (59). All models were estimated using maximum likelihood, and multiple initial values (5000 starts; 100 optimizations) were used to avoid local maxima. We imputed five datasets with an EM technique in SPSS (version 20.0) and replaced missing values by the mean of all imputed values. Mplus (version 7.0) software (64) was used for the LCA (65, 66).

After selecting a solution with an optimal number of classes, the obtained classes were compared on sociodemographic and psychosocial predictors in grade 7–8 as well as on substance-related problems the following year (grade 11). We evaluated the association between classes and each predictor with all predictors simultaneously in the model. Predictors were linked to class group membership using multinomial regression. For the outcome (attributed substance-related problems), we compared the means of the class model using equality of means test across classes based on posterior probability-based multiple imputation [AUXILIARY option in Mplus; (64)].

## Results

### Descriptive statistics

Means and standard deviation for continuous variables as well as percentages for categorical variables are presented in Table 1. Missing data ranged from 1 (age) to 776 (48%) (outcome) with a mean of 205 (12.7%).

**Table 1:** **Descriptive statistics for substance-use variables, predictors, and outcome**.

	*N*	Mean (or %)	SD
**PRÉDICTEURS (GRADE 7–8)**			
Sex (1 = female)	1578	0.53	0.49
Age	1617	0.65	0.48
Family adversity	1300	1.63	1.55
Delinquent behaviors	1377	2.60	3.45
Depressive symptoms	1345	8.68	7.48
Peer delinquency	1428	1.12	1.01
Academic achievement	1448	77.30	39.62
School Bonding	1442	3.88	1.12
Parental monitoring	1390	1.90	0.70
Conflict with parents	1398	1.27	0.62
**SUBSTANCE-USE (GRADE 10)**			
Alcohol early-onset (grade 8 or earlier)	1169	0.76	0.43
Cannabis early-onset (grade 8 or earlier)	1103	0.61	0.49
Tobacco use (non-smoker)	1618		
Occasional		13.8	
Regular		28.2	
Alcohol use frequency (0–5 times)	1448		
6–30 Times		47.2	
31 or more		14.8	
Binge drinking frequency (never)	1610		
1 or 2 times		42.0	
3 or more		25.0	
Number of drinks in typical occasion (0–3 drinks)	1603		
(4–6 Drinks)		32.3	
(More than 6)		43.3	
Cannabis use frequency (1–5 times)	1362		
6–30 Times		26.3	
31 or more		10.0	
Number of joints in typical occasion	1370	0.46	0.50
Alcohol and cannabis simultaneous use frequency (never)	1614		
1 or 2 times		38.3	
3 or more		33.4	
Stimulants/hallucinogens use frequency (never)	1601		
1 or 2 times		20.8	
3 or more		23.8	
Outcome (grade 11)	842	0.19	0.27

*SD, standard deviation*.

### Selection of latent class model

Comparisons of entropy and spillover indices, fit indices (deviance, AIC, BIC, SSBIC), and likelihood ratio tests for the one to six class LCA models suggested that the four-class model provided the best fit (see Table 2). As can be seen, model fit on all indices tended to improve as the number of classes increased, but the rate of improvement started to diminish around a four-class model. This solution had close to the lowest BIC and adjusted BIC scores with the highest entropy value of 0.83 (60). Likelihood ratio tests suggest few incremental validity beyond a four-class model. Models with 5 and 6 classes did not significantly improve model fit over models with fewer classes. The removal of covariates and outcome did not result in a change to the four-class solution, contrary to other solutions, indicating that the assumption of local independence was not violated. The four-class model also appears better than simpler models and more clinically significant. The four classes are distinct and each represents a significant number of participants. And as we will see below, classes can be discriminated by their association with predictors and outcome. We thus selected a four-class model.

**Table 2:** **Fit statistics, likelihood ratio tests, and entropy for different class solutions**.

	Fit indices				Likelihood ratio tests		Entropy	Spill
	LL	BIC	SSBIC	AIC	VLMR	Adjusted LMR		
1 Class	−45044	90361	90243	90161	NA	NA	NA	NA
2 Classes	−13095	26522	26379	26279	2837.38 (1)***	2823.731 (1)***	83	No
3 Classes	−12716	25971	25739	25578	757.79 (2)***	754.14 (2)***	83	No
4 Classes	−12477	25700	25379	25156	478.17 (3)***	475.87 (3)***	83	No
5 Classes	−12322	25598	25188	24903	308.73 (4)	307.24 (4)	81	Yes
6 Classes	−12224	25607	25109	24761	197.02 (5)	196.07 (5)	80	Yes

*LL, loglikelihood; BIC, Bayesian information criterion; SSBIC, sample-size adjusted Bayesian information criterion; AIC, Aikaike information criterion; VLMR, Vuong–Lo–Mendell–Rubin likelihood ratio test for k − 1 (H0) vs. k Classes; Adjusted LMR, Lo–Mendell–Rubin adjusted likelihood ratio test*.

***p < 0.001.

### Characteristics of the four latent classes

**Figure 1:**
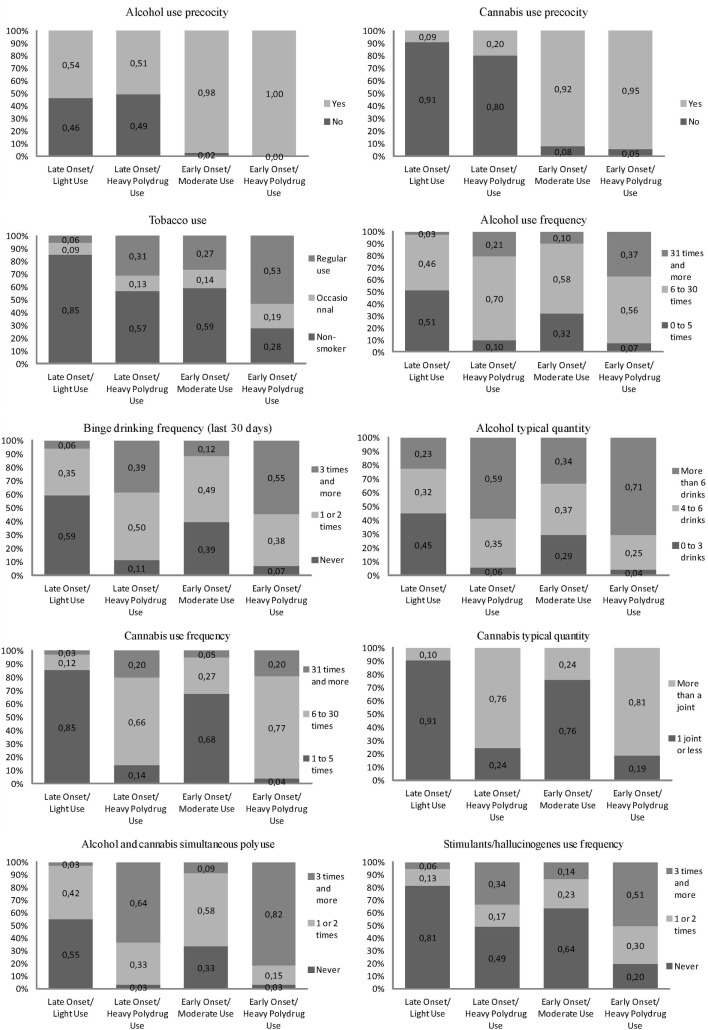
**Substance use items’ category frequencies by classes**.

Four distinct classes based on use patterns were identified. These classes were labeled Late-Light Use (1; *N* = 454, 28%), Late-Heavy + Polydrug Use (2; *N* = 222, 14%), Early-Moderate Use (3; *N* = 526, 33%), and Early-Heavy + Polydrug Use (4; *N* = 416, 26%) (see Figure 1). There are significant differences at the 0.05 level between all classes on all items except alcohol use precocity for comparisons with the Early-Heavy + Polydrug Use class, which had no variance on this item. There are differences between almost all items’ categories. Late Onset/Light Users had the lowest levels of use on each substance-related indicator. Early-Heavy + Polydrug Users had the highest levels of use on most indicators. The other two classes fell in between. Tobacco use was the highest in the Early-Heavy + Polydrug Users, the lowest in Late-Light Users, and was similar between the two other classes. For alcohol use indicators (frequency, binge, typical quantity), Early-Heavy + Polydrug Users and Late-Heavy + Polydrug Users are at similar levels despite early-onsetters showing slightly heavier patterns. Once again, Late-Light Users showed the lowest levels with Early-Moderate Users falling in between. In terms of cannabis use indicators (frequency, typical quantity) as well as of stimulant/hallucinogens and of cannabis use and alcohol polyuse, we observe very similar patterns.

### Substance-related problems outcome

As shown in Table 4, on a mean scale of the 14 substance-related harm items, scores were respectively 0.09, 0.26, 0.17, and 0.36 for each class and were all mutually statistically different. As expected, the Early-Heavy + Polydrug Use class had the highest levels of problems (*M* = 0.36, SD = 0.012) and the Late-Light Use the lowest (*M* = 0.09; SD = 008). Notably, the Early-Moderate Use class had a lower level of problems (*M* = 0.17; SD = 009) than the Late-Heavy + Polydrug Use (*M* = 0.26; SD = 0.017).

### Sociodemographic, behavioral, and psychosocial predictors of substance-use classes

As shown in Tables 3 and 4, significant differences were found between all classes. Odds ratios are calculated for a one standard deviation variation in predictors. Classes Late-Heavy + Polydrug Use and Early-Heavy + Polydrug Use were similar in terms of concurrent use in grade 10. However, compared to the Late-Heavy + Polydrug Use class, Early-Heavy + Polydrug Users had more problems in grade 11, and were older, had an earlier substance use onset (alcohol and cannabis), had a higher proportion of boys, more delinquent behaviors, deviant peers, and conflict with parents and were less monitored by them. Early-Moderate Use and Early-Heavy + Polydrug Use classes both are early-onsetters, but compared to the Early-Heavy + Polydrug Use class (and Late-Heavy + Polydrug Use), the Early-Moderate Use class had more moderate patterns of use as reflected in less problems than in Late-Heavy + Polydrug Use or Early-Heavy + Polydrug Use classes. The Early-Moderate Use class had the highest proportion of female. It has lower peer deviancy proportions than Early-Heavy + Polydrug Use class, but higher than Late-Light Use and Late-Heavy + Polydrug Use classes. It also has comparable levels of delinquent behaviors and parent monitoring with the Early-Heavy + Polydrug Use class, which are at more problematic levels than in Late-Light Use and Late-Heavy + Polydrug Use classes. The Late-Light Use class had, in addition to the lowest level of problems, the lowest level of substance use as well as the lowest level of risk. This class is younger than all other three, it had higher levels of school bonding than both Late-Heavy + Polydrug Use and Early-Heavy + Polydrug Use classes, and it had the lowest peer deviancy but had similar levels of delinquent behaviors with the Late-Heavy + Polydrug Use class, which were lower than in Early-Moderate Use and Early-Heavy + Polydrug Use classes. A similar pattern emerged regarding parental monitoring. The Late-Light Use and Late-Heavy + Polydrug Use classes had similar levels, which were higher than in Early-Moderate Use and Early-Heavy + Polydrug Use classes. The Late-Light Use class had also lower levels of conflicts than the Early-Heavy + Polydrug Use.

**Table 3:** **Adjusted associations between psychosocial predictors (grade 7–8) and class membership**.

	Class membership, OR (95% CI)					
	Reference = late-onset-light-users			Reference = late-onset-heavy-poly-users		Reference = early-onset-moderate-users
	Late-onset-heavy- poly-users	Early-onset- moderate-users	Early-onset-heavy- poly-users	Early-onset- moderate-users	Early-onset-heavy- poly-users	Early-onset-heavy- poly-users
**SOCIODEMOGRAPHIC PREDICTORS**						
Sex (1 = female)	0.58 (0.46, 0.72)***	1.93 (1.46, 2.57)***	1.36 (1.03, 1.80)*	3.36 (2.42, 4.65)***	2.36 (1.70, 3.29)***	0.70 (0.58, 0.86)***
Age	0.95 (0.79, 1.14)	1.44 (1.18, 1.76)***	1.37 (1.11, 1.68)**	1.52 (1.21, 1.91)***	1.44 (1.14, 1.83)**	0.95 (0.81, 1.12)
Family adversity	1.00 (0.78, 1.26)	1.18 (0.94, 1.48)	1.22 (0.98, 1.52)	1.19 (0.91, 1.56)	1.23 (0.94, 1.61)	1.03 (0.89, 1.20)
**BEHAVIORAL AND PSYCHOSOCIAL PREDICTORS**						
Delinquency	0.94 (0.56, 1.56)	4.82 (2.98, 7.82)***	5.21 (3.20, 8.51)***	5.15 (2.98, 8.89)***	5.56 (3.20, 9.68)***	1.08 (0.90, 1.29)
Depressive symptoms	1.01 (0.77, 1.33)	1.08 (0.82, 1.42)	1.12 (0.86, 1.45)	1.06 (0.76, 1.49)	1.10 (0.78, 1.55)	1.04 (0.87, 1.24)
Peer delinquency	1.55 (1.04, 2.30)*	6.44 (4.71, 8.79)***	8.47 (6.11, 11.75)***	4.16 (2.61, 6.64)***	5.48 (3.44, 8.72)***	1.32 (1.08, 1.60)**
Academic achievement	0.96 (0.77, 1.20)	0.93 (0.74, 1.16)	1.12 (0.97, 1.30)	0.96 (0.77, 1.20)	1.16 (1.00, 1.35)	1.16 (0.93, 1.45)
School bonding	0.81 (0.66, 1.00)*	0.84 (0.67, 1.04)	0.80 (0.63, 1.01)	1.03 (0.80, 1.32)	0.98 (0.75, 1.29)	0.96 (0.81, 1.13)
Parental monitoring	1.05 (0.84, 1.31)	0.74 (0.58, 0.95)*	0.73 (0.57, 0.93)*	0.71 (0.52, 0.95)*	0.69 (0.51, 0.94)*	0.98 (0.82, 1.18)
Conflict with parents	1.00 (0.78, 1.28)	1.28 (0.99, 1.65)	1.35 (1.05, 1.73)*	1.27 (0.94, 1.73)	1.35 (0.99, 1.82)	1.06 (0.89, 1.25)

***p < 0.001; **p < 0.01; *p < 0.05.

**Table 4:** **Adjusted outcomes at age 16 of substance-use classes**.

	Estimated means			
	Late-onset- light-users	Late-onset-heavy- poly-users	Early-onset-moderate- users	Early-onset-heavy- poly-users
Attributed substance-related problems	0.093	0.257	0.174	0.364

## Discussion

This study aimed to identify distinct latent classes of adolescent cannabis users based on their substance-use patterns in grade 10 and to distinguish these classes in terms of (1) sociodemographic and psychosocial predictors in grades 7–8 and (2) substance-related problems in grade 11. We identified four classes of cannabis use in adolescence: (1) Late-Light Use (2) Late-Heavy + Polydrug Use (3) Early-Moderate Use, and (4) Early-Heavy + Polydrug Use. Past typologies have generally found three or four cannabis users categories (5–8). However, some of these often relied on clinical samples (40) or adult population (5), whereas the current study examines a normative population of adolescents. As in other typologies, we found early and late onset classes. In general, Early-Heavy + Polydrug Users had the scores associated with the greatest risk in early adolescence and reported the most problems in late adolescence. The category with least problems and risk was the Late-Light Use class. These two classes at the extremes of the continuum differed on almost every substance use indicators and predictors.

One major contribution of the present study was to distinguish between two types of early-onset classes. Interestingly, the Early-Moderate use class had an early alcohol and cannabis use onset as in the Early-Heavy + Polydrug use class but has less substance-related problems. It even has fewer problems than the Late-Heavy + Polydrug use class, which has a late onset but heavier use patterns. This suggests that proximal substance use behavior has an influence on the level of problems experienced obviously. Except for age and sex, the only variable to distinguish between early-onset classes is peer delinquency, and between the late-onset classes is school bonding. Indeed, the Early-Heavy + Polydrug use class shows higher scores of peer delinquency than the Early-Moderate use class and the Late-Heavy Polydrug use has lower school bonding scores than the Late-Light use class. Moreover, despite possibly contributing to delaying onset (it is higher in both late onset classes), parental monitoring is no panacea because early-onset classes are high and indistinguishable on that characteristic while showing an important difference in substance-related harm. Regarding the two intermediary classes (Late-Heavy + Polydrug use and Early-Moderate use), noteworthy are the lower delinquency and peer delinquency as well as higher parental monitoring scores in the Early-Moderate use class that has a generally lower level of use and consequences. Adolescents in the Early-Moderate use class may well be the popular ones [see Ref. (67)]. The inclusion of multidimensional predictors was useful to discriminate between classes. Indeed, age, sex, delinquent behaviors, peer deviancy, school bonding, and parental monitoring all contributed to discriminate classes. The inclusion of multiple substance use indicators seems to have also improved the discrimination between classes.

### Implications

The results have many implications. First, they discriminate two different types of early as well as late onset cannabis users. They do so by shedding light on their distinctive relationships with risk factors from multiple dimensions as well as substance-related problems. Furthermore, our results, taken together, also shed light on the fact that not all early-onsetters are at elevated risk and experience a high level of substance-use related problems and that they are even at a lower level of risk than some late-onsetters. These results should be used to more meaningfully target and inform effective interventions toward users experiencing elevated levels of risks and harms. Moreover, a typology provides a useful heuristic for clinicians conducting assessment or screening with cannabis-involved adolescents. Our results suggest that screening and intervention for risky cannabis use among adolescents should focus on school bonding in order to discriminate late-onset classes and on peer delinquency in order to discriminate early-onset classes. Intervention should be prioritized for the Early-Heavy + Polydrug use and Late-Heavy + Polydrug use classes. School bonding and peer deviancy seem to be good targets for intervention with either Early- or Late-Heavy + Polydrug Users and parental monitoring and conflict with parents seem to be further good targets for intervention with Early-Heavy + Polydrug users. In both cases (Early- or Late-Heavy + Polydrug), binge drinking, cannabis use frequency and alcohol and cannabis simultaneous use seem to be the most important substance-use behaviors to target in interventions. In the first case, working simultaneously on these use patterns, in addition to stimulants/hallucinogens use frequency, and psychosocial risk factors, with demand and harm reduction interventions, would probably be a good strategy whereas intervention with the latter group should focus primarily on use patterns. Indeed, it is noteworthy and important to take into account that the Late-Heavy + Polydrug use class is mostly constituted of females with lower levels of risk. These cannabis users are more difficult to predict, but they have important intervention needs. The Early-Moderate Users would on their part benefit from early intervention strategies in order to prevent their use to shift from moderate to heavy as well as to prevent it to become more problematic. Overall, other than substance use behaviors, the main factors to target would generally be school bonding, delinquency, peer delinquency, and parental monitoring. In terms of policy implications, the current legal framework in Canada and elsewhere is characterized by the criminalization of all use; any cannabis use is defined as problematic (68). This approach differs from the one prevailing for alcohol, which has evolved to a public health framework (69, 70). Rather than focusing on use *per se*, priority is given to the risks and harms associated with problematic patterns of use (e.g., drunk driving). This way, targeted interventions may be applied to relevant behaviors (71). In our study, this could mean targeting binge drinking frequency and substance mixing behaviors as well as other substance use. Harm reduction strategies also seem to be potential useful tools in order to reduce cannabis-related problems.

### Strengths and limitations

This study has multiple strengths, including the simultaneous consideration of substance use severity indicators, predictors, and outcomes as well as their multidimensionality, and use of a large prospective community-based sample. However, this study is not without limitations. First, despite the fact that confidentiality was assured, response bias and common method variance could have influenced our results. Fortunately, the validity and reliability of self-reported data on substance use have been established (72–74), but this has not been proved for self-report of problems. In addition, the sample comes from deprived areas, which is a limitation to the generalization of results. However, even if schools from deprived areas were sampled, individual scores of familial adversity vary and include participants from low familial adversity. Another limitation is related to the large amount of missing data and potential attrition bias. Also, the results do not provide information on the sequence of problem as well as the subgroup development over time. Finally, the inclusion of age of onset in the typology, while substance use indicators have been selected from grade 10 is another potential limitation to the current study.

### Future studies

Future prospective studies should examine factors that explain transitions across these subtypes in time. This would however be complex because age of onset is included in the typology. Another important area of development is in the study of specific harm categories (relational, health, school, etc.) related to different patterns of use in order to better inform prevention and treatment efforts to target specific harms. Indeed, if different outcomes are related to different classes, intervention should not only target specific factors related to specific patterns but also focus on specific problems related to each. Which are the most important problems related to each class? Which classes are disproportionally represented for each problem? Another potential improvement over the current study is the use of more specific items for each other drugs than alcohol, Tobacco, and Cannabis (e.g., ecstasy, LSD, Speed, GHB, Ketamine, etc.) as well as substance use motives. Finally, a nationally representative sample would also improve the external validity of the typology.

## Conflict of Interest Statement

The authors declare that the research was conducted in the absence of any commercial or financial relationships that could be construed as a potential conflict of interest.
